# Clinical observation using virtual reality for dental education on surgical tooth extraction: A comparative study

**DOI:** 10.1186/s12909-024-05605-w

**Published:** 2024-06-07

**Authors:** Yiseul Choi, Myoungho Lee, Jaeyeon Kim, Wonse Park

**Affiliations:** 1 Department of Advanced General Dentistry, Yonsei University College of Dentistry, Seoul, Korea; 2 Institute for Innovation in Digital Healthcare, Yonsei University, Seoul, Korea

**Keywords:** Virtual reality, Clinical observation, Dental education, Dental student, Surgical education, Tooth extraction

## Abstract

**Background:**

Clinical observation conducted during the 3rd and 4th years of dental school is an important part of dental students’ clinical education. However, conventional clinical observation is associated with several problems, including the lack of opportunity for all students to assist during surgery. Virtual reality (VR) technologies and devices can be used to demonstrate clinical processes that dental students need to learn through clinical observation. This study aimed to evaluate the effectiveness of teaching dental students the surgical tooth extraction procedure through clinical observation using VR.

**Methods:**

We recruited third- and fourth-year dental students and divided them into a VR clinical observation group (VR group) and a conventional clinical observation group (control group). The control group visited an outpatient clinic and observed an oral and maxillofacial specialist perform surgical tooth extraction, whereas the VR group watched a 360° video of surgical tooth extraction using a head-mounted display. After observation, both groups were surveyed regarding their satisfaction with the clinical observation and their understanding of the procedure.

**Results:**

Understanding of the procedure and satisfaction with the observation were significantly higher in the VR group than in the control group (*p* = 0.001 and *p* = 0.047, respectively). Compared with conventional clinical observation, VR clinical observation improved learning motivation and medical thinking and judgment skills; however, interaction between professors and students was lacking.

**Conclusions:**

VR clinical observation using 360° videos might be an effective teaching method for students. However, to allow interaction between professors and students during clinical observations, using it along with conventional clinical observation is necessary.

## Background

Clinical observation or attendance is a crucial part of dental education in the third and fourth years of dental school. The most significant difference between dental and medical clinical education is that, unlike medical students, dental students begin treating patients in hospital clinics during their undergraduate years. To provide patient care in hospitals, it is essential for students to observe patient treatments in a real clinical setting. Therefore, clinical observation is introduced as the first step for dental students when they begin clinical rotations in their third year of dental school [1]. During clinical observation, students not only learn about treatment procedures but also focus on communication, patient interaction, and assisting during clinical procedures. Learning through assisting is particularly important for dental procedures such as minor oral surgeries and extractions, as it allows students to understand how the operator performs the procedure.

The most effective clinical observation and training often involves students assisting experienced clinicians who are also educators, preferably professors [2]. However, clinical observation has some limitations. First, assistance during professor-led treatments is usually provided by dental residents or assistants. Therefore, not all students get an opportunity to assist during surgery, and students often spend their time observing the procedure from a distance without being able to see inside the oral cavity. This passive observation can limit students’ abilities to gain a clear understanding of treatment procedures, especially in a confined environment such as the oral cavity. Second, students in the early stages of learning may lack familiarity with assisting tasks such as using a suction device, which can lead to friction between the operator and student. Additionally, when focusing solely on assisting during a procedure, students may find it challenging to grasp important aspects, such as the progression of the procedure, rationale behind specific techniques, difficulty level of the surgery, and key points of the procedure.

Recently, advances in Virtual Reality (VR) and Augmented Reality (AR) technologies have enabled their widespread use for medical education [3–5]. Platforms within VR environments enable the demonstration of surgical procedures across various medical fields using three-dimensional (3D) images and touchscreens [6, 7]. AR applications are used to teach technical operative skills [8, 9]. In addition, platforms are available for anatomical education through virtual 3D images [10]. Furthermore, instead of using virtual 3D images, surgical scenes are captured using 360° cameras to create VR content for training medical practitioners [11, 12]. The content can be viewed using VR educational tools such as head-mounted displays (HMDs) that are fixed on the user’s head using straps and provide an immersive virtual reality experience.

Clinical observation using VR could help dental students improve their understanding of the surgical process. In addition, VR with 360° video allows students to check not only the surgical process but also patient interactions, which could be a tool to increase satisfaction with clinical observation. This study aimed to assess the effectiveness of demonstrating the surgical extraction procedure, which is typically learnt through clinical observation, to dental students using VR technology and HMDs.

## Methods

### Participants

This study included third- and fourth-year dental students from the Yonsei University College of Dentistry who were capable of wearing an HMD and watching VR content for at least 10 min. Individuals who found it difficult to wear HMDs, those with poor visual acuity that made it challenging to watch VR content, and those who were unable to read the survey questions were excluded.

The sample size was calculated using G-Power 3·1·9·2, with an effect size of 0·5, a significance level of 0·05, a statistical power of 80%, and Df of 1, considering a dropout rate of 20% in the Chi-square test [13]. The study participants were divided into two groups: the VR observation group (VR group) and the conventional observation group (control group). A total of 40 participants were recruited, with 10 third-year and 10 fourth-year students assigned to each group. The recruited participants were divided by grade and randomly assigned to each group using block randomization with a block size of four.

#### 360° videos

**Fig. 1:**
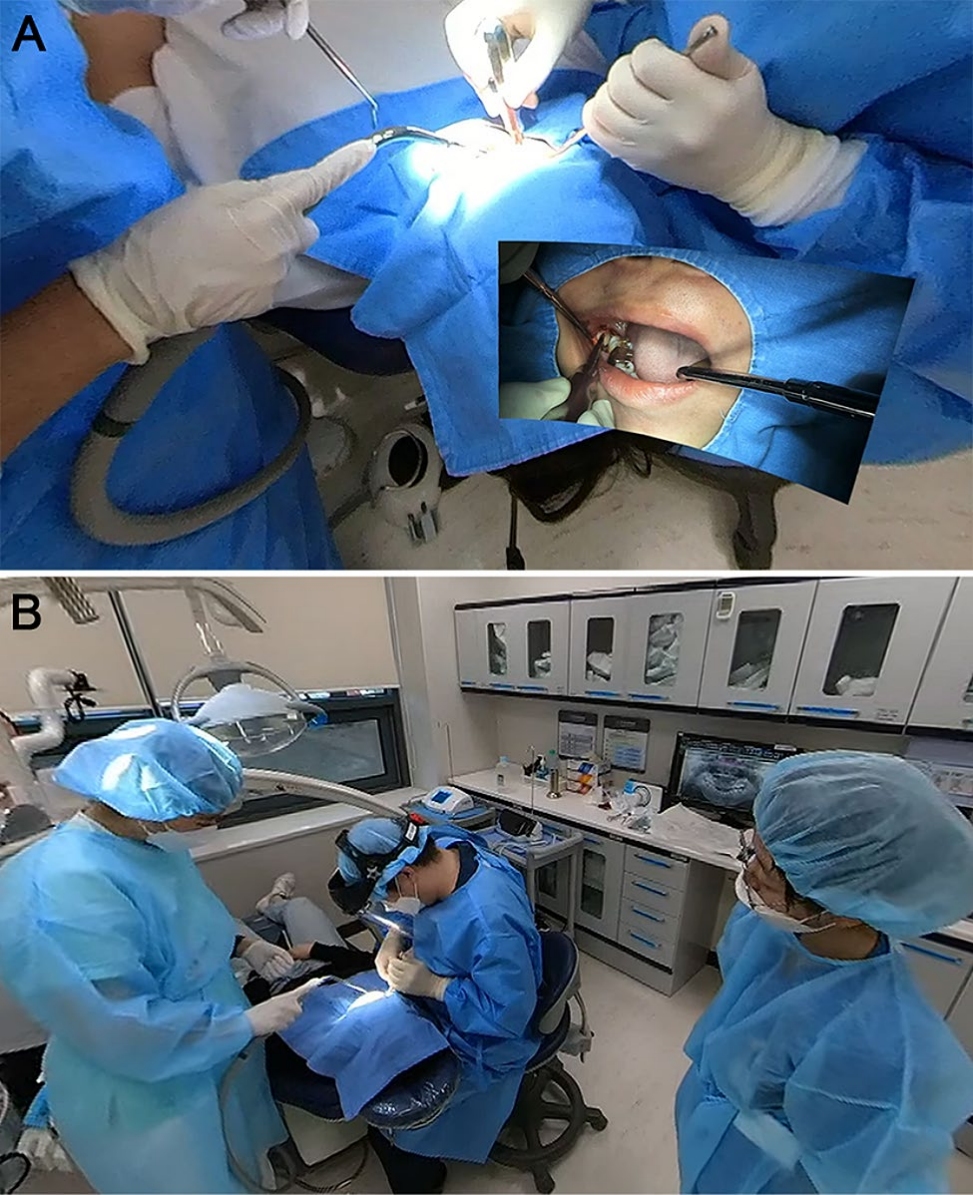
Clinical observation method for the tooth extraction used in the study (**A**) A scene from the 360° video used in the study. The overall interior of the operating room was filmed using a 360°camera (One X2, Insta360, CA, USA), and the patient’s oral cavity was filmed using a miniature dental camera (ProCam XS, Futudent, Helsinki, Finland). Depending on the observer’s field of view, other objects in the operating room might also be visible.; (**B**) A conventional clinical observation method. An observer observed the surgery from the back of the patient’s head in the operating room

We used a 360° camera (One X2, Insta360, CA, USA) and a miniature dental camera (ProCam XS, Futudent, Helsinki, Finland) to film the overall interior of the operating room and the patient’s oral cavity, respectively, during surgical extraction of a mandibular third molar at the Pell and Gregory classification level B, class II, and with mesioangular or horizontal inclination according to Winter’s classification [14, 15]. The filmed footage was edited to ensure that patient identification data, including patient number and name, were not visible. The footage was stitched using Insta Studio software (Insta360, CA, USA) and then the 360° and flat videos were overlaid using Adobe Premiere Pro (Adobe Stock, CA, USA). The final 360° videos used in the study was provided at 30 frames per second and a resolution of 3,840 × 2,160 pixels. The total video playback time was 13 min, and the surgical process from incision to suture was provided in videos. The 360° videos were recorded from the assistant’s position with a view of the entire operating room, while the flat videos were recorded inside the patient’s mouth. Because it was a recording of a typical mandibular third molar surgical extraction process, there was no interactive contents for the VR viewer (Fig. 1A).

### Observation method

Participants in the control group visited the Department of Advanced General Dentistry at Yonsei University Dental Hospital individually according to the patient’s schedule for conventional clinical observations. Similar to the 360° video, they observed surgical extractions of mandibular third molars meeting the criteria of class II, level B according to the Pell and Gregory classification, and mesioangular or horizontal inclination according to Winter’s classification on panoramic radiographs. (Fig. 1B) Identical operating rooms were used for all clinical observation, and all surgeries were performed by the same oral and maxillofacial surgeon as the 360° videos. Clinical observations were conducted from the start of the surgery until suturing.

VR observation was conducted at the time desired by the participants in a room with no obstacles, and all lights turned off. Participants wore an HMD (MetaQuest 2, Meta, CA, USA) and watched a 360° video of the surgical extraction of an impacted mandibular third molar filmed in the operating room where conventional clinical observations were conducted.

Both groups completed a 25-question questionnaire after clinical observation. The questionnaire used in the study was developed with reference to the study by Lee et al. [16]. The following data were obtained: (1) VR clinical observation experience; (2) opinions on the observation method used in the study compared to conventional clinical observation methods; (3) procedural understanding and satisfaction; (4) intention to recommend VR clinical observation; (5) Advantages and disadvantages of VR clinical observation; and (6) clinical observation methods desired in future training courses. Procedural understanding was assessed by considering whether the surgical process and instrument operation method were clearly visible and understood in the clinical observations that participated in the study. Satisfaction was assessed by comprehensively considering the time spent on clinical observation, understanding of the procedure, and visibility. Survey responses were recorded on a 5-point scale.

### Statistical analysis

Data were statistically analysed using IBM SPSS (version 25.0; IBM Corporation, NY, USA). Reliability analysis was performed to determine whether the participants provided consistent responses. The Shapiro–Wilk test was used to assess all continuous variables for conformity with a normal distribution. The Mann–Whitney U test was performed to compare opinions regarding the observation methods used in the study, including satisfaction with the clinical observation methods and understanding of the procedure, between the groups. In addition, the Kruskal–Wallis test was performed further compare procedural understanding and satisfaction according to the clinical observation method and grade or clinical observation method and gender. Data are presented as frequencies (%), as all variables are categorical. Statistical significance was set at *p* < 0.05.

## Results

A total of 40 students participated in the survey. Their general characteristics are presented in Table 1. The questionnaire was found to be reliable based on a Cronbach’s alpha of 0.861.

**Table 1:** General characteristics of participants (*n* = 40)

Characteristics	VR group (*n* = 20)	Control group (*n* = 20)
Age (yr)	23.80 ± 1.79	25.25 ± 2.63
Sex (*n*) Female Male	7 13	7 13
Grade (*n*) 3rd -year 4th -year	10 10	10 10
Observation time (minutes) Tooth extraction site (*n*) #38 #48	13.00 0 20	12.94 ± 3.52 8 12

**Fig. 2:**
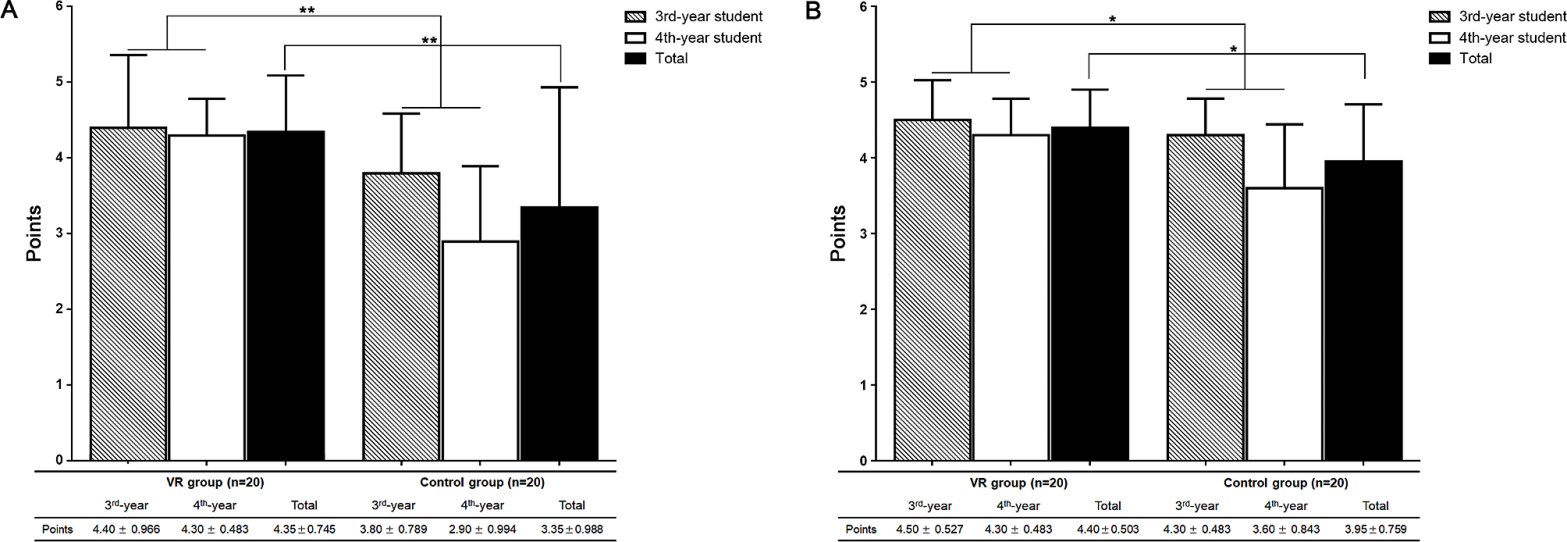
Comparison of scores according to clinical observation method (**A**) Comparison of procedural understanding. Procedural understanding was significantly different between the groups, with mean scores of 3.35 ± 0.988 and 4.35 ± 0.745 points in the control and VR groups, respectively (*p* = 0.001). When comparing procedural understanding according to the grade and clinical observation method, significant differences were found between the groups (F = 13.488, *p* = 0.004).; (**B**) Comparison of satisfaction with observation. Satisfaction with observation was significantly different between the groups, with mean scores of 4.40 ± 0.503 and 3.95 ± 0.759 points in the control and VR groups, respectively (*p* = 0.047). When satisfaction was compared between grades and clinical observation methods, a significant difference was observed between the groups (F = 8.676, *p* = 0.034). Data are presented as means ± SD. *p < 0.05, **p < 0.005

No participant had previous experience with VR clinical observation. Procedural understanding was significantly different between the groups, with mean scores of 3.35 ± 0.988 and 4.35 ± 0.745 points in the control and VR groups, respectively (*p* = 0.001). When comparing procedural understanding according to the grade and clinical observation method, significant differences were found between the groups (F = 13.488, *p* = 0.004). In the VR group, 3rd -year students scored 4.40 ± 0.966 points and 4th -year students scored 4.30 ± 0.483 points, and in the control group, 3rd -year students scored 3.80 ± 0.789 points and 4th -year students scored 2.90 ± 0.994 points (Fig. 2A).

Satisfaction with observation was significantly higher in the VR group than in the control group (4.40 ± 0.503 vs. 3.95 ± 0.759 points; *p* = 0.047). When satisfaction was compared between grades and clinical observation methods, a significant difference was observed between the groups (F = 8.676, *p* = 0.034). In the VR group, satisfaction was 4.50 ± 0.527 points for 3rd -year students and 4.30 ± 0.483 points for 4th -year students. In the control group, 3rd -year students scored 4.30 ± 0.483 points and 4th -year students scored 3.60 ± 0.843 points. The largest difference and most significant difference in satisfaction was observed between the 3rd -year VR and the 4th -year control groups (4.50 ± 0.527 vs. 3.60 ± 0.843 points; *p <* 0.005) (Fig. 2B).

**Fig. 3:**
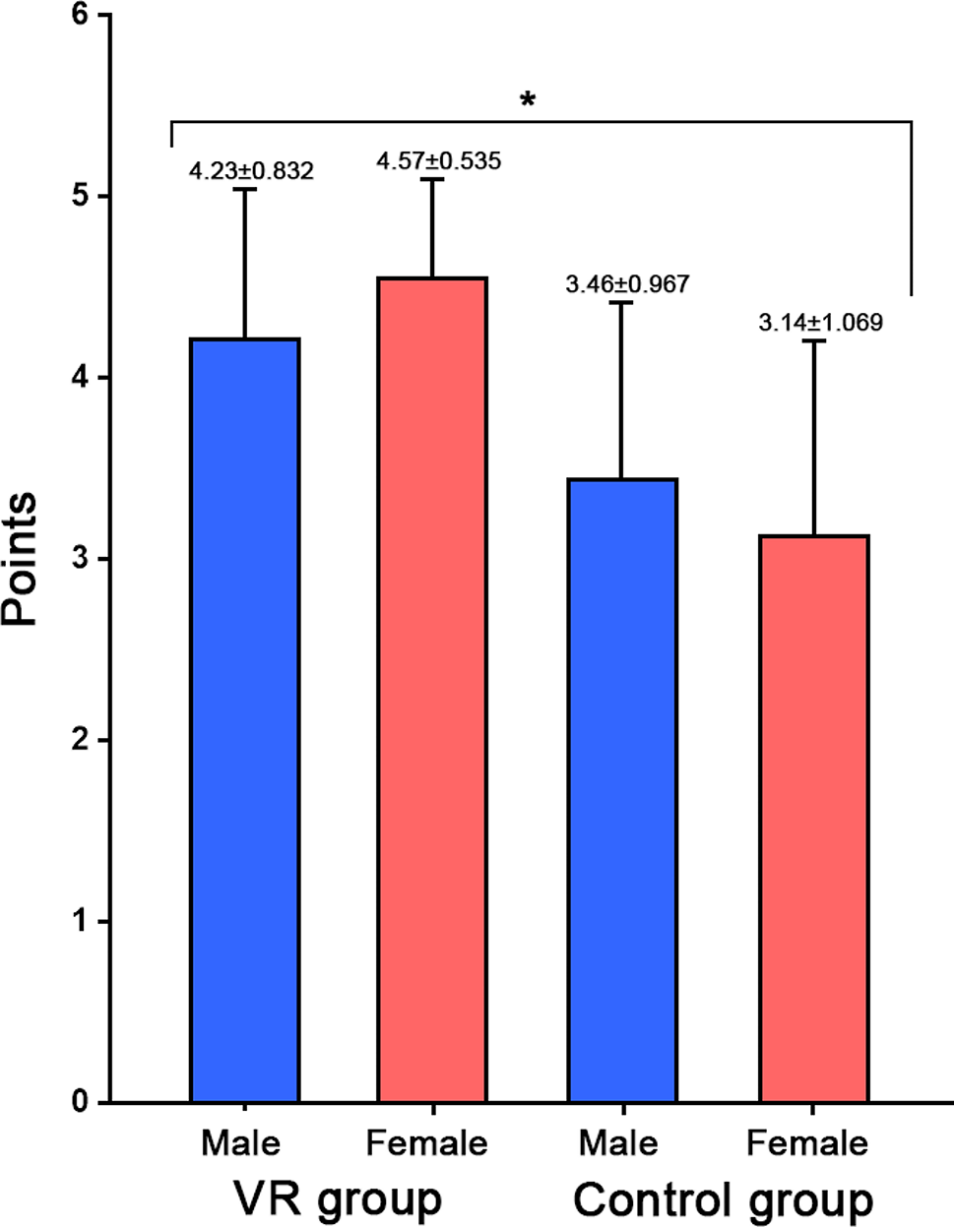
Comparison procedural understanding according to the gender and clinical observation method When comparing procedure understanding according to gender and clinical observation method, there was a significant difference between groups (F = 4.692, *p* = 0.007). In the VR group, men scored 4.23 ± 0.832 points and women scored 4.57 ± 0.535 points, and in the control group, men scored 3.46 ± 0.967 points and women scored 3.14 ± 1.069 points. Data are presented as means ± SD. *p < 0.05

As a result of comparing procedural understanding according to gender and clinical observation method, there was a significant difference between groups (F = 4.692, *p* = 0.007). Women in the VR group had the highest score of 4.57 ± 0.535 points, and women in the control group had the lowest score of 3.14 ± 1.069 points (Fig. 3). When comparing satisfaction according to gender and clinical observation method, there was no significant difference between groups.

When comparing previous clinical observation experiences with the observation method used in this study, VR clinical observation was found to induce significantly more learning motivation than conventional clinical observation. In addition, VR clinical observation led to more active participation than conventional clinical observation and was more helpful in achieving learning goals (Table 2).

**Table 2:** Comparison of clinical observation experience and observations participated in the study

Question	VR group (*n* = 20)	Control group (*n* = 20)	*p*-value
Compared to previous clinical observations, observations in the study			
induced learning motivation.	4.25 ± 0.639	3.60 ± 0.883	0.015
induced more active participation in observation.	4.45 ± 0.686	3.80 ± 0.894	0.016
had a positive influence on thinking and behaviour.	3.95 ± 0.826	3.60 ± 0.883	0.190
enabled lively exchange between professors and students.	2.85 ± 1.089	3.10 ± 0.852	0.327
developed ability to respond to actual clinical situations.	3.95 ± 1.099	3.70 ± 1.081	0.421
enhanced medical thinking and judgment skills.	4.15 ± 0.745	3.40 ± 10.95	0.025
had an appropriate overall level.	4.55 ± 0.510	4.10 ± 0.852	0.083
provided an overall observation time.	4.70 ± 0.470	3.95 ± 1.099	0.015
were easy to understand.	4.60 ± 0.503	3.70 ± 0.733	< 0.001
helped achieve learning goals.	4.60 ± 0.598	3.45 ± 1.050	< 0.001

**Fig. 4:**
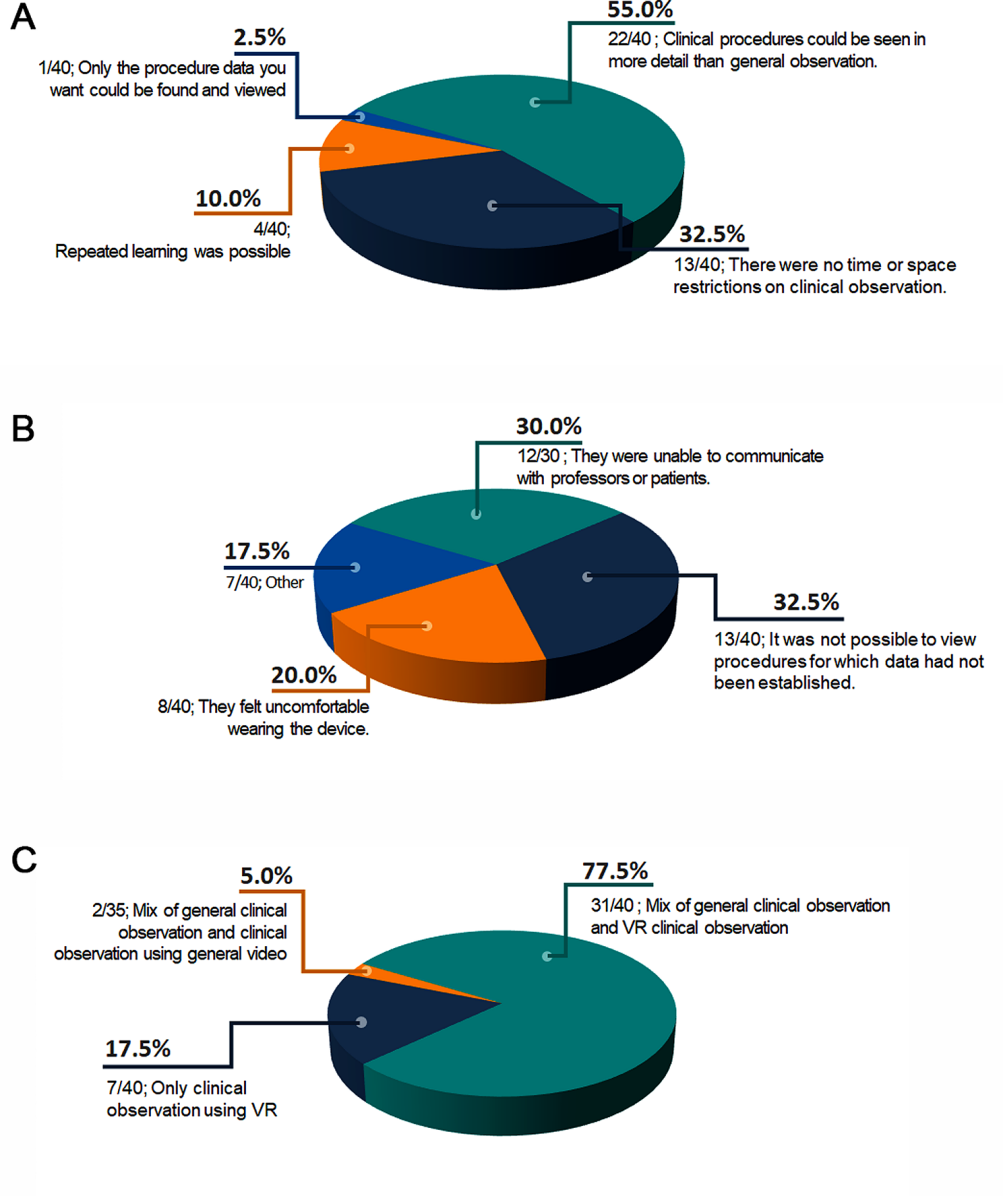
Participants’ survey results (**A**) Advantages of clinical observation using VR; (**B**) Disadvantages of clinical observation using VR; (**C**) Preferred clinical observation method for future training courses

Willingness to recommend ‘clinical observation using VR’ to others was expressed by 39 out of 40 participants, with no differences between the groups. Regarding the advantages of clinical observation using VR, the most frequent response (55.0%) was that clinical procedures could be seen in more detail than in conventional observations. Other answers were as follows: There were no time or space restrictions on clinical observation (32.5%), repeated learning was possible (10.0%), and only the desired procedure data could be found and viewed (2.5%). Regarding the disadvantages of clinical observation using VR, the most frequent response (32.5%) was the inability to view procedures for which data had not been established. In addition, 30.0% of participants responded that they were unable to communicate with the professors or patients, and 20.0% felt uncomfortable wearing the device. Finally, regarding the desired clinical observation method for future training courses, 77.5% of respondents preferred combining conventional and VR clinical observation, whereas 17.5% of respondents preferred clinical observation using VR alone (Fig. 4).

## Discussion

### Principal results

In this study, we compared the efficacy of conventional and VR clinical observation for dental education. Students showed high satisfaction with observing surgical tooth extraction using VR, and their understanding of the surgical procedure improved. In terms of satisfaction and understanding according to the clinical observation method, 3rd -year students in the VR group had the highest scores, and 4th -year students in the control group had the lowest scores. This was similar to the results of previous studies on education using VR, and the lack of experience in the operating room seemed to have influenced the education method using VR [12].

Observation methods that use VR offer various advantages to students. VR provides a realistic simulation and enables a clear understanding of the surgical process through detailed 3D visual representations, which improves student satisfaction. However, VR has some drawbacks. Students responded that real-time communication with professors or patients was difficult during clinical observations using VR. These limitations can cause discomfort because of the lack of interaction in a realistic surgical environment.

Therefore, based on the students’ responses, a combination of conventional and VR clinical observations might provide the best educational effect. Conventional observation provides students with the experience of communicating with real patients and professors, and a sense of the actual surgical environment. In contrast, VR observation helps increase students’ understanding through safe and enhanced visualization. This approach provides students with various learning experiences and helps maximize their learning outcomes.

### Comparison with prior work

Digital applications are widely used in the field of dental education. Several studies have reported that VR education is effective [17, 18]. Correa et al. used VR and haptic devices for dental local anaesthesia training and reported satisfactory outcomes [19]. Al-Saud et al. showed that novices made fewer errors when they received both expert and VR simulator feedback and suggested that expert guidance and VR should be combined during the early stages of training [20]. In our study, participants in the VR group had high levels of understanding and learning satisfaction. However, the fact that a mix of conventional clinical and VR clinical observation methods was the most preferred approach suggests that expert guidance is also important for beginners.

Pulijala et al. used a combination of an HMD and 360° video to train oral and maxillofacial surgeons and reported that VR surgical videos are useful visualization aids and practice-based learning tools for surgical trainees [21]. Our study also used 360° video and HMD to allow dental students to have the same clinical experience as in reality. After observing the tooth extraction surgery through VR, participants showed high satisfaction with and understanding of the surgery. This is consistent with the findings in previous studies, and confirms that VR can be used as an effective tool in surgical education.

In this study, we compared procedure understanding to see if there were differences according to gender and clinical observation method, and women in the VR group showed the highest understanding. Some studies have also reported that women showed improved cognitive abilities or better performance in VR compared to men. When assessing visuospatial reasoning in virtual reality via a HDM, women performed better than men [22]. Allen et al. reported that women performed better in VR cognitive tasks, and Liang et al. reported that women performed better than men in memory tasks [23, 24]. Additionally, when using VR teaching methods, female students showed favorable performance than male students in both empathy and actual behaviors [25]. These findings might be due to the fact that women demonstrate higher levels of empathy and motivation to learn compared to men [26].

However, there were differences between our study and previous studies. As preclinical dental education is important for dental students, several studies have focused on the effects of simulator education using haptics [19, 27, 28]. In contrast, the VR content used in our study focused on an immersive observation educational method while wearing an HMD. Although direct skill improvement cannot be expected, this is a useful method for mastering dental treatment procedures and learning the instruments used in each process.

### Limitations

This study had several limitations. First, a crossover design was not applied. Because our study had a small study population, it would have been more efficient to apply a crossover design. Participants performed only conventional or VR observation according to the group allocation. Therefore, a direct comparison of the differences in students’ actual surgical education experiences could not be performed. Second, all students participating in the study observed the surgical procedures of one surgeon. However, differentiation between surgeons could provide insight into potential variability and bias in outcomes. It will be necessary to provide students with conventional or VR clinical observation of the surgical procedures of two or more different surgeons for comparative evaluation. Third, our study relied primarily on students’ evaluations and did not include more objective measurement indicators, such as evaluations by external experts or comparisons with actual surgical outcomes. Further research is required to improve these areas and to assess a wider range of learning outcomes.

These limitations might affect the interpretation and generalization of our results. However, they can be used as important information to suggest future research directions and improvements. Further studies with a crossover design to compare students’ actual experiences, considering wearability and convenience, and focusing on improving technology for real-time communication are required to develop more effective teaching methods.

## Conclusions

VR clinical observation using 360° video might be an effective educational method for dental students. Our results can serve as an important reference for educators considering new teaching methods in the field of dental education. However, for exchanges between professors and students during clinical observations, a combination of VR and conventional clinical observations is necessary.

## Data Availability

Data that support the findings of this study are available from the corresponding authors upon request and following IRB rules and privacy regulations.

## Abbreviations

HMD: Head-mounted display
VR: Virtual reality
AR: Augmented Reality
3D: Three-dimensional
